# The Role of Neopterin and Interleukin‐6 Detection in Saliva and Plasma in Oral and Oropharyngeal Cancer Patients: A Prospective Study

**DOI:** 10.1002/cre2.70202

**Published:** 2025-08-19

**Authors:** Lenka Šašková, Peter Tvrdý, Bohuslav Melichar, Josef Tomandl, Jana Zapletalová, Michal Mozol'a, Petr Michl, David Král, Richard Pink

**Affiliations:** ^1^ Department of Oral and Maxillofacial surgery, Faculty of Medicine and Dentistry Palacky University Olomouc and University Hospital Olomouc Czech Republic; ^2^ Department of Oncology, Faculty of Medicine and Dentistry Palacky University Olomouc and University Hospital Olomouc Czech Republic; ^3^ Department of Biochemistry, Faculty of Medicine Masaryk University Brno Czech Republic; ^4^ Department of Medical Biophysics, Faculty of Medicine and Dentistry Palacky University Olomouc Olomouc Czech Republic

**Keywords:** interleukin‐6, neopterin, squamous cell carcinoma of head and neck

## Abstract

**Objectives:**

The incidence of oral and oropharyngeal cancer is continually rising and affects increasingly younger patients. Consequently, many studies focus on early diagnosis using appropriate biomarkers. Neopterin and interleukin‐6 (IL‐6) are promising predictive and prognostic markers of immune response activation, both systemic and local, due to the anatomical proximity of malignancies to the salivary glands.

**Material and Methods:**

We collected oral fluid samples from 50 patients before and after the surgical resection of squamous cell carcinoma of the oral cavity and oropharynx. Additionally, blood samples were withdrawn from 20 of these patients and levels of neopterin and IL‐6 were estimated using ELISA commercial kits. All gathered data were subsequently statistically analyzed for evaluation and compared to values from a control group of healthy individuals.

**Results:**

In patients with oral squamous cell carcinoma (OSCC) and oropharyngeal squamous cell carcinoma (OPSCC), there was a significant decrease in neopterin and IL‐6 levels in saliva following the surgical removal of the malignancy. These postoperative levels approached those of the control group. There was no significant decrease in neopterin and IL‐6 levels in plasma.

**Conclusion:**

Detection of neopterin and IL‐6 in saliva is a reliable diagnostic method for early detection of OSCC and its recurrence, as well as for monitoring therapeutic success, compared to plasma. Neopterin and IL‐6 appear to be promising prognostic and predictive markers of the disease.

## 1 Introduction

Oral and oropharyngeal malignancies represent the 6th most common cancer subsite globally (Rahman et al. 2020). Histologically, they are predominantly squamous cell carcinomas (95%), followed by malignant tumors of glandular epithelium (adenocarcinomas, mucoepidermoid carcinomas), and less frequently by sarcomas, lymphomas, melanomas, or distant metastases from other sites (Pazdera 2022). In 2020, there were 747,316 new cases of lip, oral cavity, and pharyngeal malignancies worldwide. This disease accounts for 4.1% of all cancer cases, and 3.7% of cancer deaths are attributable to malignancies in these locations (Sung et al. 2021). The incidence is steadily increasing, which may be attributed not only to increased exposure to risk factors (tobacco, alcohol, betel, HPV, stress, poor dietary habits, etc.) but also to early detection of cancer at an earlier stage (Huang et al. 2023). Early diagnosis can be significantly aided by the examination of biomarkers.

A sufficiently specific prognostic and predictive biomarker for mucosal oral and oropharyngeal squamous cell carcinomas (OSCC and OPSCC, respectively) that meets practical criteria has not yet been identified. Most studies published so far have focused on biomarkers related to tumor cell properties, while biomarkers of the host's immune response have generally been overlooked (Melichar 2013). With the increasing use of immunotherapy as a treatment strategy, attention is now shifting toward biomarkers of host immune system activation, among which neopterin and interleukin‐6 are included (Melichar et al. 2017; St. John et al. 2004).

## 2 Neopterin

Neopterin is a derivative of guanine, belonging to the pteridine group, and is naturally present in body fluids. It is synthesized in human monocytes/macrophages and dendritic cells. Neopterin is considered an indicator of T lymphocyte and macrophage activation. Its increased production is part of the immune response to infection, inflammation, autoimmune diseases, and finally, yet importantly, tumor growth (Hoffmann et al. 2003). Elevated levels of neopterin may also be associated with prognosis and therapeutic response in certain oncological diagnoses. Neopterin levels can be measured in various biological fluids, such as blood, urine, cerebrospinal fluid, and oral fluid, using specific analytical techniques such as enzyme‐linked immunosorbent assay (ELISA) or high‐performance liquid chromatography (Hamerlinck 1999).

## 3 Interleukin‐6

Interleukin‐6 (IL‐6) is an inflammatory cytokine produced by several types of cells, predominantly T‐lymphocytes, macrophages, and stromal cells, in response to stimulation by tumor necrosis factor‐alpha (TNF‐α) and interleukin‐1 (IL‐1). The activated IL‐6 complex plays a role in cellular differentiation, proliferation, and apoptosis. IL‐6 influences the course of chronic inflammation, thus creating a favorable tumor microenvironment and supporting the growth of malignancies (Rašková et al. 2022).

Elevated levels of IL‐6 are described in malignancies of the kidney, bladder, colorectal region, ovaries, gallbladder, pancreas, lymphatic tissue, and specifically in squamous cell carcinoma of head and neck (SCCHN) (Lacina et al. 2018). Furthermore, IL‐6 levels are typically increased in cardiovascular diseases, type II diabetes, and other conditions that activate the immune system (autoimmune diseases, graft‐versus.‐host disease (GVHD), chronic or acute infectious diseases). Its detection is possible both conventionally from blood plasma or serum and from other body fluids such as urine, cerebrospinal fluid, tears, and, notably, oral fluid (Kreiner et al. 2022; Chen et al. 1999).

## 4 Methods

Between 2013 and 2017, 50 patients with oral and oropharyngeal squamous cell carcinoma (OSCC, *n* = 44), (OPSCC, *n* = 6), respectively, were diagnosed at the Department of Oral, Maxillofacial, and Facial Surgery at the University Hospital in Olomouc, who were suitable for our study. Patients who had undergone radiotherapy or chemotherapy before or right after the surgical procedure were excluded from the study.

The aim of the study was to determine the levels of neopterin in saliva (*n* = 50) and plasma (*n* = 20), as well as IL‐6 in saliva (*n* = 20) and plasma (*n* = 20) in patients diagnosed with OSCC and OPSCC, and to compare these levels with those measured after the surgical removal of the tumor before initiating adjuvant oncological therapy in patients with advanced stages of squamous cell carcinoma of the head and neck (SCCHN). The measured values were then compared with the neopterin and IL‐6 levels in the control group (*n* = 47 and *n* = 20, respectively).

Among the 50 patients, there were 33 males (66.0%) and 17 females (34.0%). The median age of the patients was 61 years (range: 39–87 years). Twelve out of the 50 patients already had a recurrent disease. The tumors were most commonly located on the floor of the mouth (*n* = 15, 30%), the lateral edge of the tongue (*n* = 12, 24%), buccal mucosa (*n* = 6, 12%), and oropharynx (*n* = 6, 12%). According to the 7th edition of the TNM classification, 47% (*n* = 23) of patients were in stage I, 35% (*n* = 17) were in stage II, 6% (*n* = 3) were in stage III, and 12% (*n* = 6) were in stage IV. T4 carcinomas exhibiting bone invasion were of the erosive type in two cases and the infiltrative type in four cases. Histopathological grading was G1 in 37.5% of patients (*n* = 18), G2 in 22.9% (*n* = 11), and G3 in 39.6% (*n* = 19). Patients with OPSCC (*n* = 6) were tested immunohistochemically p16 negative in 5 cases and only one patient was p16 positive.

Thirty two patients (64%) achieved 5‐year overall survival from the time of initial diagnosis. By the end of the year 2023, 11 patients had died from malignancy, 10 from other causes, one patient was alive with active disease, and 28 patients were surviving without signs of disease relapse.

All patients, including those in the control group, underwent a periodontal examination using a World Health Organization (WHO) probe, which measured the probing depth of dental sulci, the presence of induced bleeding, and/or dental calculus. Based on the measured data, we determined the Community Periodontal Index of Treatment Needs (CPITN).

Informed consent was obtained from both the patients and the control group before enrollment in this study.

Patients were followed up in collaboration with the Department of Oncology.

### 4.1 Control Group

The control group consisted of 47 individuals without systemic disease, with healthy periodontal tissues (i.e., low CPITN values), with a median age of 24 years (range: 22–51 years). The age of the patient group was significantly higher than that of the control group. The median age in the control group was 24 years.

### 4.2 Sample Collection

In patients with histologically confirmed squamous cell carcinoma, we collected approximately 5 ml of unstimulated saliva according to Navazesh (Navazesh and Kumar 2008) 1 day before the OSCC excision, in a dimmed room, at least 30 min after eating and brushing teeth. After saliva collection, a periodontal examination was performed, and CPITN values were noted. Saliva samples were stored in the dark containers and frozen at −20°C, while blood samples were immediately centrifuged at 3,000 rpm for 15 min at 4°C, and plasma was stored at −70°C. Saliva samples were centrifuged in the same manner, but after thawing and immediately before the ELISA analysis. We repeated this procedure in patients approximately 1–2 months after the surgical exstirpation of SCCC, following complete wound healing and before any potential adjuvant oncological treatment. The same procedure for collecting and storing oral fluid and blood samples was repeated for the control group.

Neopterin and IL‐6 levels were detected using enzyme‐linked immunosorbent assay (ELISA ‐ Enzyme Linked Immunosorbent Assay, IBL International GMHB, Hamburg, Germany) for quantitative analysis, designed for human serum, plasma, and urine, which are also suitable for detecting neopterin and IL‐6 in saliva.

Neopterin was quantified by a competitive ELISA, in which sample antigen and enzyme‐labeled antigen compete for binding to rabbit anti‐neopterin antibodies. Antibody‐antigen complexes were captured by goat anti‐rabbit antibodies coated on the plate. The resulting color intensity was inversely proportional to the neopterin concentration and measured spectrophotometrically using a standard curve (IBL International 2012).

IL‐6 levels were measured using a sandwich ELISA. A monoclonal antibody pre‐coated on microplate wells captured human IL‐6 from samples. A biotin‐conjugated anti‐IL‐6 detection antibody and streptavidin‐HRP were added sequentially. Color development was proportional to the IL‐6 concentration and quantified by absorbance at 450 nm with reference to a standard curve (IBL International 2014).

Due to the high cost of the ELISA kits, each sample was analyzed as a single measurement, without technical duplication.

### 4.3 Data Analysis

Data analysis was conducted using IBM SPSS Statistics version 23 (Armonk, NY: IBM Corp). Correlations between changes in CPITN and changes in neopterin or IL‐6 levels in saliva were assessed using Spearman's rank correlation analysis. Spearman's correlation analysis was also used to evaluate the dependence of the measured parameters on age. Preoperative and postoperative values were compared using the Wilcoxon signed‐rank test. The Kruskal–Wallis test was employed to assess the relationship between preoperative levels of neopterin and IL‐6 levels and histopathological grading, as well as between TNM classification stages. The Mann–Whitney *U* test was used to evaluate the dependence of preoperative values on *N* classification, bone invasion, tumor duplication, recurrence, or sex. Significant predictors of survival were determined using Cox regression analysis. Receiver operating characteristic (ROC) analysis was utilized to identify the optimal cutoff value for neopterin levels in saliva postoperatively. Normality of quantitative parameters was tested using the Shapiro–Wilk test. All tests were conducted at a significance level of *p* = 0.05.

## 5 Results

**Figure 1:**
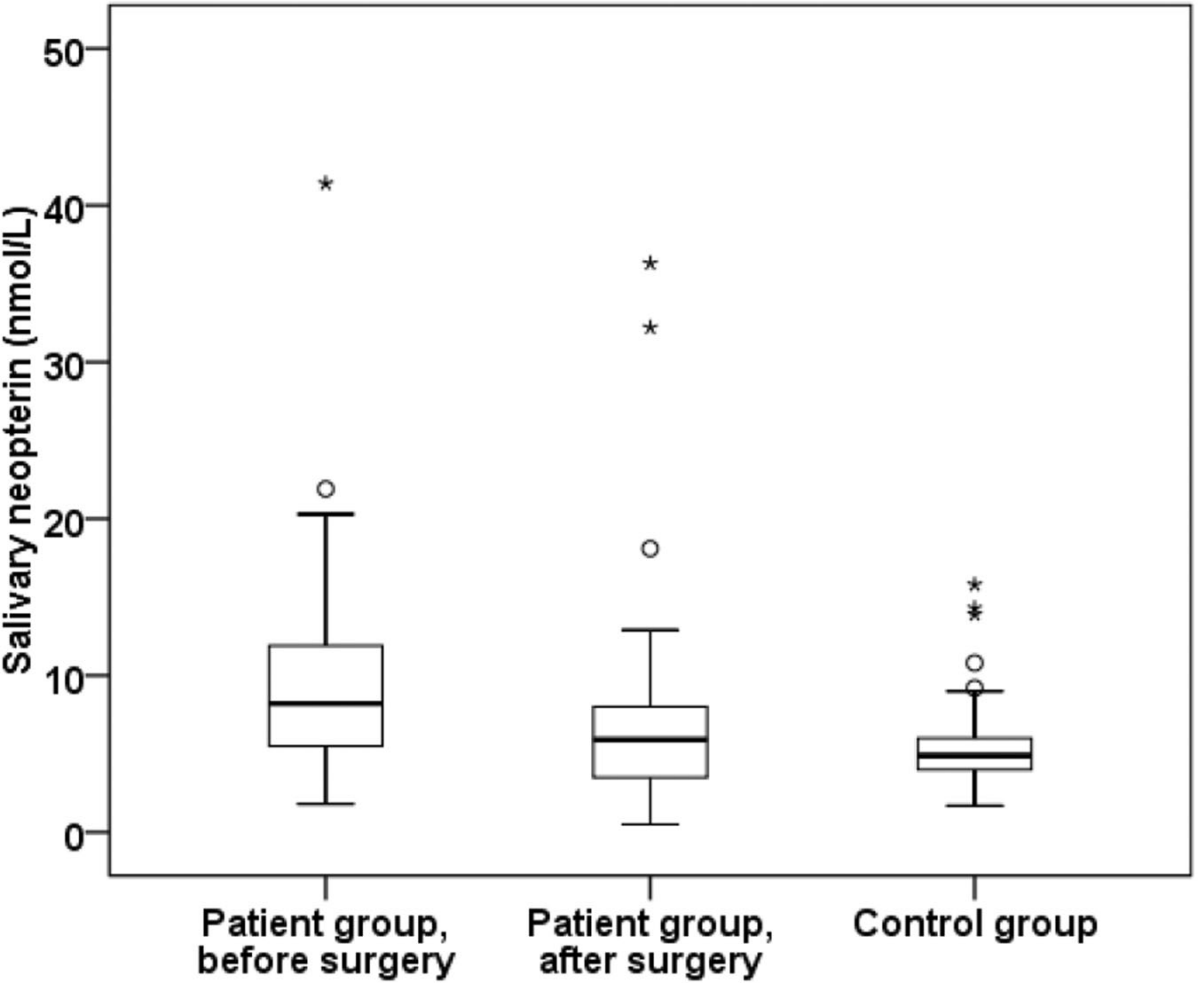
Quartile box diagram of neopterin in saliva in the patient group before/after surgery and the control group. Neopterin level after surgical removal of OSCC/OPSCC significantly decreased, and postoperative levels of neopterin approached to those of the control group.

After the surgical excision of OSCC, a significant reduction in salivary neopterin levels was observed when compared to neopterin levels after the healing of surgical wounds (the median value before surgery was 8.1 nmol/L and after surgery was 5.5 nmol/L, *p* = 0.001). Neopterin levels in oral fluid after OSCC removal approached those of the control group (Figure 1, Table 1).

**Table 1:** Comparison of salivary/plasmatic levels of neopterin and salivary levels of IL‐6 in the patient group before and after surgery.

	Patient group, presurgery	Patient group, postsurgery	Control group	Patient group pre versus post (*p* value)	Patient group pre versus control (*p* value)	Patient group post versus control (*p* value)
NEO (nmol/L) saliva	8.1 (1.8–41.4)	5.5 (0.5–36.3)	4.9 (1.7–15.8)	**0.001**	**0.0002**	0.611
IL‐6 (pg/mL) saliva	16.1 (2.1–346)	7.1 (1.7–69.1)	< 1.56	**0.049**	—	—
NEO (nmol/L) plasma	7.6 (1.5–15.4)	7.8 (1.0–20.2)	5.2 (1.3–11.3)	0.614	0.107	0.110

*Note:* Comparison of salivary/plasmatic neopterin and salivary IL‐6 in the control group and in the patient group before/after surgery. Neopterin and IL‐6 levels in saliva significantly decreased after surgical removal of OSCC/OPSCC. Bold values indicate statistically significant.

Through Receiver Operating Characteristic (ROC) analysis, an elevated level of neopterin in saliva was demonstrated as a significant parameter for predicting the presence of oral cancer, with an Area Under the Curve (AUC) value of 0.721 (*p* = 0.0002) and a cutoff value of 6.015 nmol/l (Table 2).

**Table 2:** ROC analysis for salivary neopterin and cutoff value for OSCC/OPSCC prediction.

	Cut off 6.015 nmol/L	
		95% CI
Sensitivity	70.0%	55.4%–82.1%
Specificity	76.6%	62.0%–87.7%
Positive predictive value	76.1%	61.2%–87.4%
Negative predictive value	70.6%	56.2%–82.5%
Accuracy	73.2%	55.4%–82.1%
False positivity	23.4%	12.3%–38.0%
False negativity	30.0%	17.9%–44.6%

**Figure 2:**
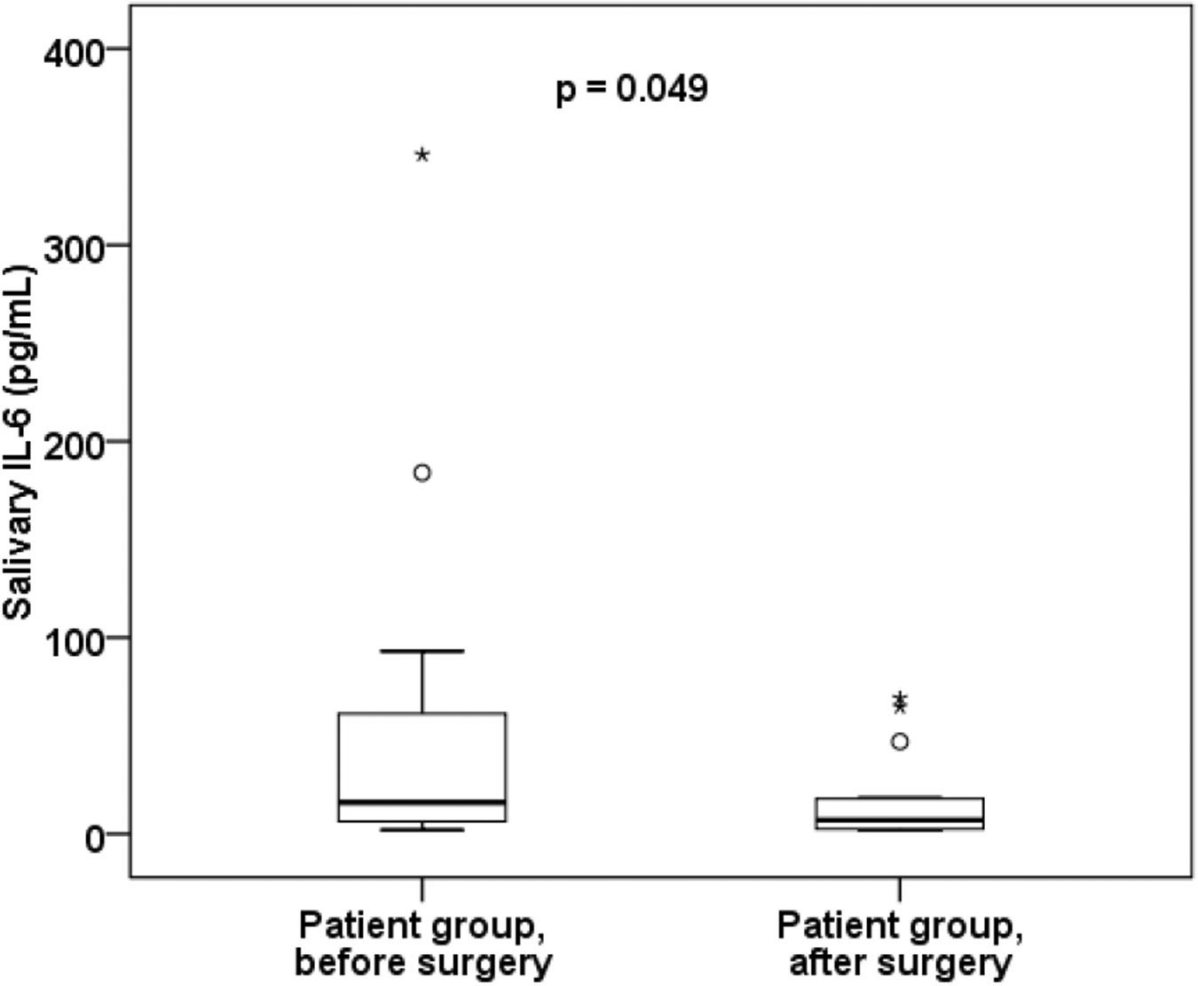
Quartile box diagram of IL/6 in saliva in the patient group before and after surgery. The level of IL‐6 in saliva significantly decreased after surgical removal of OSCC/OPSCC.

Additionally, a significant decrease in salivary IL‐6 levels was demonstrated postoperatively (median before surgery was 16.1 pg/ml and after surgery was 7.1 pg/mL, *p* = 0.049) (Table 1, Figure 2). As the levels of salivary IL‐6 in the control group were mostly under the sensitivity borderline of the ELISA kit (lower detection limit is < 1.56 pg/mL), ROC analysis was not possible; thus, the cutoff value for salivary IL‐6 was not calculated.

The plasma levels of neopterin did not decrease on average following the removal of OSCC or OPSCC (Table 1). In most cases, IL‐6 plasma levels were below the sensitivity threshold of the ELISA kit for IL‐6 detection even before the surgical removal of the tumor. Therefore, it is not statistically feasible to assess any potential decrease or increase in IL‐6 levels.

**Figure 3:**
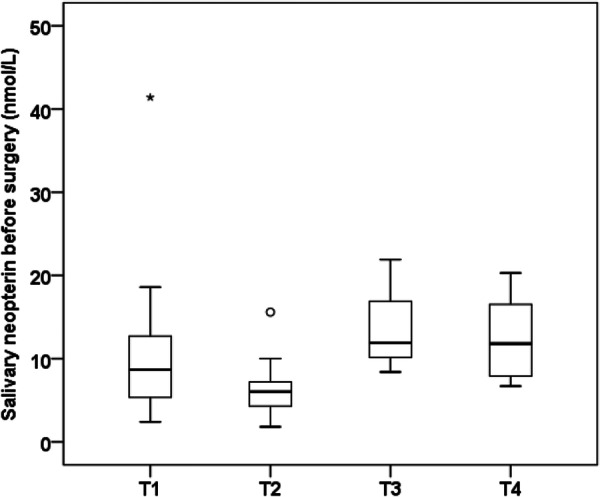
Quartile box diagram of neopterin‐level distribution in saliva before surgery related to size (t‐classification) of an OSCC/OPSCC; preoperative levels of salivary neopterin were significantly lower in T1 and T2 tumors than in T3 and T4 tumors, *p* = 0.011.

Histopathological grading did not significantly correlate with preoperative neopterin levels in saliva (*p* = 0.264) or plasma (*p* = 0.636), nor IL‐6 levels in saliva (*p* = 0.304) and plasma (Table 3). A significant association was demonstrated between tumor (T) classification and preoperative salivary neopterin levels (*p* = 0.011) (Figure 3). In the case of classification T3 and T4, higher levels of preoperative neopterin in saliva were measured when compared to patients with T1 and T2 tumors. No significant relationship was found between T classification and preoperative plasmatic neopterin (*p* = 0.953) and salivary/plasmatic IL‐6 levels (*p* = 0.304) (Table 3).

**Table 3:** Correlation of clinicopathological data with levels of salivary/plasmatic neopterin and salivary IL‐6 before and after surgery.

		NEO saliva presurgery	IL‐6 saliva presurgery	NEO plasma presurgery
Grading	G1	11.1 (2.4–41.4)	4.9 (2.7–7.1)	9.0 (8.4–9.6)
	G2	6.0 (1.8–14.8)	10.1 (2.1–39.0)	7.6 (1.5–8.7)
	G3	7.9 (2.8–21.9)	17.8 (3.8–346)	7.5 (4.6–15.4)
	*p*‐value	*p* = 0.264	*p* = 0.097	0.636
T	T1 + T2	7.1 (1.8–41.4)	10.1 (2.1–184)	7.6 (1.5–15.4)
	T3 + T4	11.8 (6.7–21.9)	61.2 (3.6–346)	8.4 (4.6–12.5)
	*p*‐value	**0.011**	0.069	0.710
N	N0	7.6 (1.8–41.4)	12.7 (2.4–184)	8.6 (4.6–15.4)
	N1 + N2	8.2 (4.2–21.9)	28.4 (2.1–346)	5.4 (1.5–7.7)
	*p*‐value	0.423	0.536	**0.012**
Bone invasion	yes	11.8 (6.7–20.3)	75.6 (14.8–93)	9.7 (4.6–12.3)
	no	7.2 (1.8–41.4)	10.6 (2.1–346)	7.5 (1.5–15.4)
	*p*‐value	0.083	0.153	0.596

No significant association was found between neopterin in saliva and IL‐6 levels in saliva and plasma and the presence or absence of nodal metastases. Surprisingly, the level of neopterin in plasma before surgery was significantly lower in the presence of nodal metastases (median: 5.4 with metastases vs. 8.6 without nodal metastases, *p* = 0.012) (Table 3).

Additionally, there was no significant relationship between preoperative levels of salivary/plasmatic levels of neopterin and salivary IL‐6, respectively, and the presence of bone invasion; however, the number of patients with bone invasion was low (*n* = 6). (Table 3).

No significant differences in neopterin and IL‐6 levels before surgery were observed in relation to recurrence and/or locoregional second primary cancer. The average survival time span from the first diagnosis (overall survival) was 90 months (95% CI: 78–102 months). The average time of disease‐free survival was 79 months (95% CI: 66–93 months), with a median time to recurrence of 99 months (95% CI: 41–157 months). Significant negative predictors of survival, identified using multivariate Cox regression analysis, included bone invasion (RR = 4.004), recurrence (RR = 3.966), and postoperative salivary neopterin levels ≤ 4.55 (RR = 2.506). The Stepwise Forward method was used to identify significant predictors.

## 6 Discussion

Saliva is a suitable diagnostic medium for several reasons. Its collection is easy, noninvasive, and safe. Compared to blood collection, saliva sampling reduces the risk of infection transmission between patients and healthcare personnel. It requires no specialized equipment and can be performed almost anywhere and repeatedly, making it an attractive alternative to other bodily fluids. Saliva contains a wide range of biomolecules, including enzymes, hormones, antibodies, microorganisms, and DNA. These biomarkers can provide information on overall health, specific diseases, infections, hormonal balance, and genetic predispositions. Many systemic diseases and pathological conditions (such as diabetes, HIV, cardiovascular diseases, certain cancers, autoimmune diseases, GVHD, and many others) can manifest in saliva. As a result, saliva can be used to monitor the effects of therapy and disease dynamics (Pink et al. 2009).

In comparison to blood or other bodily fluids, saliva is easier to store and transport, simplifying the logistics of testing. Due to these advantages, saliva is becoming an increasingly popular diagnostic tool in various fields of medicine and research. The benefits of its collection are also evident in this prospective study.

When comparing neopterin levels in saliva and plasma, as well as IL‐6 levels in saliva and plasma, no significant correlation was observed. This result suggests that the level of local immune system activation in the tumor microenvironment is higher than the activation of systemic cellular immunity. Tumor‐associated macrophages may play a crucial role in neopterin synthesis (Pittet et al. 2022); thus, concentration in saliva is higher than in serum and significantly changes after tumor excision. In case of IL‐6, cancer‐associated fibroblasts, tumor infiltrating lymphocytes, and/or tumor‐associated macrophages in the tumor microenvironment explain higher levels of IL‐6 in saliva and its drop after tumor removal (Raudenska et al. 2023; Koontongkaew 2013).

In the statistical evaluation of salivary neopterin and IL‐6 levels, we observed a significant decrease following surgical tumor excision, whereas neopterin and IL‐6 levels in the blood were not significantly altered and were considerably lower, making them more difficult to detect compared to saliva. Given the variability of neopterin and IL‐6 levels in patients both before and after surgery, it is clearly more appropriate to repeat saliva sampling and compare results over time. Additionally, monitoring and comparing neopterin and IL‐6 levels in high‐risk patients and those with premalignant conditions is recommended.

Several prospective studies have revealed a relationship between oral health and the activation of both local and systemic cellular immune systems. For example, Vrecko et al. demonstrated an increase in salivary neopterin levels in patients with periodontitis (Vrecko et al. 1997). Neopterin levels in urine, which reflect systemic activation of the cellular immune system, were only mildly elevated. Thus, neopterin detection may serve as a useful tool for monitoring the presence, activity, or progression of periodontal disease, but it cannot distinguish between chronic periodontitis and its aggressive form (Gottumukkala et al. 2022).

Despite the findings of similar studies (Vrecko et al. 1997; Heneberk et al. 2022), we did not observe a correlation in our patient group between changes in CPITN (Community Periodontal Index of Treatment Needs) after surgery and changes in salivary neopterin or IL‐6 levels. To our best knowledge, we did not find any previous studies that measured the dynamics of neopterin and IL‐6 together with periodontal health examination.

In CPITN (Community Periodontal Index of Treatment Needs) assessments, there is potential for subjective influence on the degree of periodontal involvement by the clinician, as inappropriate or insufficient pressure may be applied during periodontal probing. In our study, a single clinician examined the majority of patients. The recommended pressure for periodontal probing is 20 to 25 g, which should be sufficient to detect inflammation and pocket depth without causing tissue damage, iatrogenic bleeding, or deeper probing, thus avoiding falsely elevated CPITN values.

There are some limitations present in our study. One of these is the significant age difference between the control group and the patient group. However, periodontal condition naturally deteriorates with age, and finding individuals with completely healthy periodontium, overall good health, and within a similar age group is quite challenging. Additionally, plasma IL‐6 levels tend to increase with age due to the prevalence of comorbidities and aging itself, which leads to higher inflammation and musculoskeletal wear (Lacina et al. 2018). This phenomenon further supports the suitability of saliva as a diagnostic medium for OSCC.

When detecting IL‐6 levels in serum, the levels were very low, and in most cases, both in the postsurgical patient group and the control group, they were below the detection threshold of the ELISA kit. Although more sensitive ELISA kits for IL‐6 detection are available, their cost is significantly higher. Once again, saliva proved to be a better diagnostic medium than plasma, as salivary IL‐6 levels were 2–3 times higher than plasma levels.

Neopterin and IL‐6 appear to be suitable primarily as markers for therapeutic efficacy or early‐stage cancer diagnosis, as suggested by their elevation in oral premalignant conditions and stage I of SCCHN (Juretić et al. 2013; Oshin et al. 2024). Our results indicate that neopterin and IL‐6 are useful, especially when used repeatedly and in combination with other potential biomarkers.

To our best knowledge, there are no similar previous prospective studies comparing salivary neopterin before and after surgical treatment, except for our own pilot study (Pink et al. 2016). There is only one study comparing salivary IL‐6 in head and neck cancer patients before and after surgical treatment, which supports the results of this study outcome (Sato et al. 2015).

## Author Contributions

**Lenka Šašková:** conceptualization, investigation, visualization, methodology, writing – original draft, data curation, project‐administration, resources. **Peter Tvrdý:** methodology, writing – review and editing, data curation, resources, conceptualization. **Bohuslav Melichar:** conceptualization, methodology, supervision. **Josef Tomandl:** methodology, data curation, formal analysis, validation. **Jana Zapletalová:** methodology, formal analysis, writing – review and editing. **Michal Mozol'a:** data curation, project‐administration. **David Král:** data curation, conceptualization, validation. **Petr Michl:** data curation, investigation, visualization. **Richard Pink:** supervision, resources, validation.

## Ethics Statement

The Ethics Committee of Palacky University approved our study. All experimental methods complied with the Helsinki Declaration.

## Consent

All participants provided written informed consent before enrollment.

## Conflicts of Interest

The authors declare no conflicts of interest.

## Data Availability

The data that support the findings of this study are available from the corresponding author upon reasonable request.
